# Entrainment of circadian rhythms to irregular light/dark cycles: a subterranean perspective

**DOI:** 10.1038/srep34264

**Published:** 2016-10-04

**Authors:** Danilo E. F. L. Flôres, Milene G. Jannetti, Veronica S. Valentinuzzi, Gisele A. Oda

**Affiliations:** 1 Institute of Biosciences, Department of Physiology, University of São Paulo; São Paulo, São Paulo, 05508-900; Brazil; 2 Centro Regional de Investigaciones Científicas y Transferencia Tecnológica de La Rioja (CRILAR), Provincia de La Rioja, UNLaR, SEGEMAR, UNCa, CONICET. Entre Ríos y Mendoza s/n, (5301) Anillaco, La Rioja, Argentina

## Abstract

Synchronization of biological rhythms to the 24-hour day/night has long been studied with model organisms, under artificial light/dark cycles in the laboratory. The commonly used rectangular light/dark cycles, comprising hours of continuous light and darkness, may not be representative of the natural light exposure for most species, including humans. Subterranean rodents live in dark underground tunnels and offer a unique opportunity to investigate extreme mechanisms of photic entrainment in the wild. Here, we show automated field recordings of the daily light exposure patterns in a South American subterranean rodent, the tuco-tuco (*Ctenomys* aff. *knighti* ). In the laboratory, we exposed tuco-tucos to a simplified version of this natural light exposure pattern, to determine the minimum light timing information that is necessary for synchronization. As predicted from our previous studies using mathematical modeling, the activity rhythm of tuco-tucos synchronized to this mostly simplified light/dark regimen consisting of a single light pulse per day, occurring at randomly scattered times within a day length interval. Our integrated semi-natural, lab and computer simulation findings indicate that photic entrainment of circadian oscillators is robust, even in face of artificially reduced exposure and increased phase instability of the synchronizing stimuli.

Synchronization of circadian rhythms to the 24-hour day/night has long been studied with model organisms under laboratory conditions, using artificially controlled light/dark (LD) cycles^1^,^2^. In mammals, this synchronization is mediated by a neuronal retino-hypothalamic pathway that transduces the light information and entrains the master circadian oscillator in the suprachiasmatic nuclei of the hypothalamus^3^. In most cases, patterns of photic entrainment are studied under “rectangular” LD cycles comprising hours of continuous light and darkness. Although this procedure has offered great insights into synchronization mechanisms, it has often been criticized because, under natural conditions, most organisms, including humans, are not continuously exposed to light during the day^4^,^5^,^6^,^7^,^8^,^9^.

An insightful approach to evaluate how much artificial LD cycles can reliably represent natural light/dark cycles has been the study of entrainment patterns under discrete and continuous, cyclic light regimens. This distinction led to the development of two conceptual models of photic entrainment, namely the “parametric” (continuous) and “non-parametric” (discrete) mechanisms, which have greatly helped to coordinate our understanding of photic synchronization^2^,^10^,^11^,^12^.

Subterranean rodents offer a unique opportunity to investigate natural photic entrainment mechanisms. On one hand, they live mostly underground in the extreme photic environment of constant darkness. On the other hand, they do expose themselves to light at least while removing earth out of their burrows; and this is true even for those species that are considered “strictly” subterranean^13^. Cumulative evidence reveals that they possess functional circadian oscillators and intact neuronal pathways for photic entrainment^14^,^15^,^16^. Furthermore, our group reported a Phase Response Curve (PRC)^17^ to light pulses for a subterranean rodent, revealing similar photic entrainment properties to those of non-subterranean rodent species^18^. The frequency of exposure to the external light should vary among subterranean rodent species but there is still little knowledge on how often and when they perform this typical behavior.

We present the first automated recording data of daily light exposure patterns in a subterranean rodent species of Argentina, the tuco-tucos (*Ctenomys* aff. *knighti*)^19^, carrying light sensing loggers^20^ in their natural habitat. Together with behavioral observations of these solitary animals^21^ the current data confirm that tuco-tucos are exposed to light at irregular times, in brief episodes of foraging and earth removal. This exposure pattern should however sustain entrainment, because individuals that were transferred directly to constant conditions upon capture from the wild displayed 24-hour period “aftereffects” of entrainment^21^.

We have previously simulated photic entrainment using a mathematical model of circadian oscillator that displayed a qualitatively similar PRC of the tuco-tucos^18^. The oscillator was given simulated daily light pulses, scattered randomly within a daytime window, to grossly model the natural daily light exposure of tuco-tucos. We manipulated the length of the daytime window and verified the limits of entrainment to this simplified model. For short time windows, entrainment was expected, as the light schedules would resemble a single-pulse T-cycle with period equal to 24 hours. As the window was widened to longer durations, the oscillator might either free-run or become arrhythmic. Our computer simulations unexpectedly predicted that entrainment to a 24-hour period might be achieved even if the light regimen were composed of one single light pulse per day, occurring at random times scattered at a broad (up to 14-hour) range along the days^18^.

In the present work, we tested the predictions experimentally with tuco-tucos in the laboratory, using wheel-running circadian rhythms as markers of their circadian oscillator motion. The confirmed synchronization under this peculiar light regimen reveals that photic entrainment is achieved by the contribution of both “parametric” and “non-parametric” effects and this is based on integrated field, laboratory and computer simulation approaches.

## Results

### Light exposure patterns during winter in semi-natural enclosures

**Figure 1:**
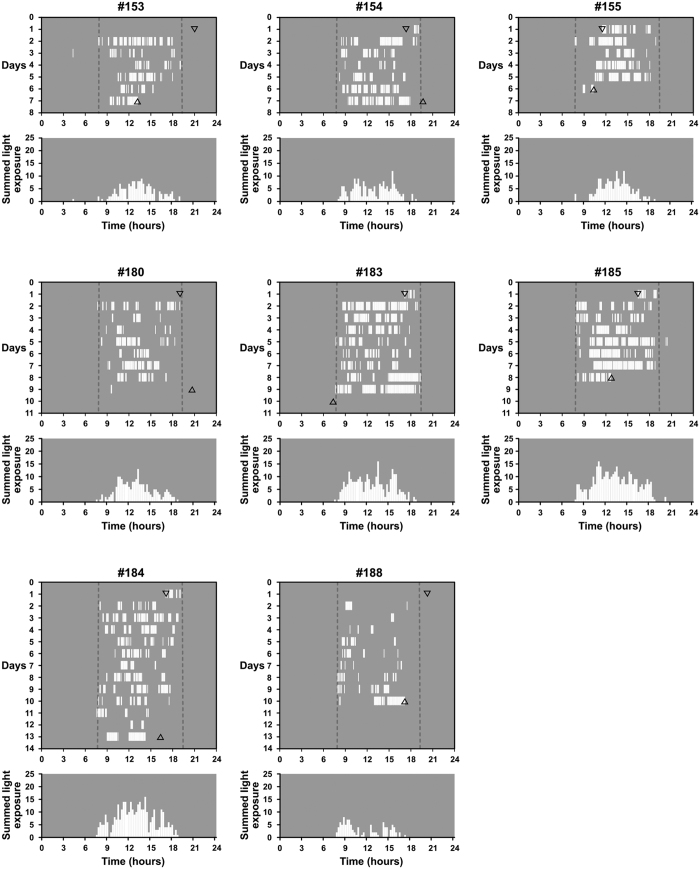
Daily temporal pattern of light exposure from 8 tuco-tucos, during the 2014 (July) and 2015 (July and August) south hemisphere winters. For each animal an actogram and a histogram illustrate its light exposure pattern. Actograms (upper graphs) show times of daily light exposure episodes (white vertical bars against a gray background) and consecutive days are shown below each other. Civil twilight times are demarked with vertical dashed lines. Downward and upward pointing triangles indicate the times of release and recapture from the enclosure, respectively. Histograms (lower graphs) show the summed light exposure of all days in the field enclosure for each animal, throughout the 24 hours of the day. Animals #153-155 were recorded in July 2014 and #180-188 in July 2015.

We recorded the daily light exposure patterns from 8 tuco-tucos maintained in field enclosures in the winters of 2014 and 2015 (Fig. 1). All animals exposed themselves to light at least once a day, in brief episodes. Within each individual record, the timing of these episodes changed from day to day. Values greater than 1.2 lux were never detected beyond the limits of civil twilight, indicating no exposure to natural or artificial light at night. Maximum recorded light levels were always close to the higher limits of detection (19,000 lux or 74,000 lux depending on the logger). It is noteworthy that even though light exposure episodes were scattered throughout the day, the overall exposure during winter was distributed in a unimodal pattern, concentrated in the middle of the day (Fig. 1).

### Entrainment under pulse regimens of increasing dispersion

The light exposure pattern recorded in the field inspired a laboratory model that captures some essential properties of the natural light/dark regimen of tuco-tucos. The model is a simplification that explores an extreme scenario with minimal timing information provided by the light/dark cycle. The protocol consists of a single light pulse of 1 hour (1,000 lux) applied once a day, at random times within a pre-defined time window “**I**”. Computer simulations of a mathematical oscillator have predicted the synchronizing potential of this simplified light exposure^18^.

**Figure 2:**
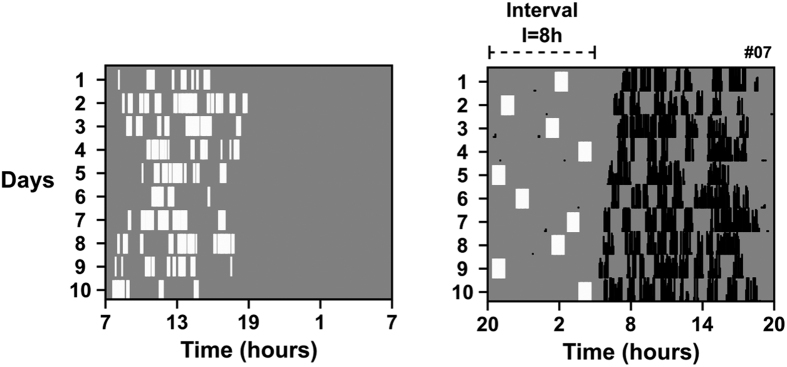
A simplified model of the tuco-tuco’s light exposure pattern. Left: Temporal pattern of light exposure in the field, measured by light sensors. Right: Light exposure model and its effect on the rhythm of a representative animal in the laboratory. The figure depicts the random dispersion of the daily light pulses (1 hour; 1,000 lux) along a fixed interval I. In both graphs, light exposure is marked in white over the darkness (gray) background. The running-wheel activity of the tuco-tuco is represented by black marks. The number on the upper right of the actogram indicates the custom lab ID of the animal.

Figure 2 illustrates the overall features of the protocol in comparison to the natural light exposure of tuco-tucos. In the example (Fig. 2 right panel), the single 1-hour light pulse was administered every day at a different time within an 8-hour interval (**I** = 8 h). Data from a representative animal illustrate that, despite the irregular light/dark timing, the activity rhythm remained stably adjusted to a 24-hour period. We describe below further results with these randomly distributed light pulses.

**Figure 3:**
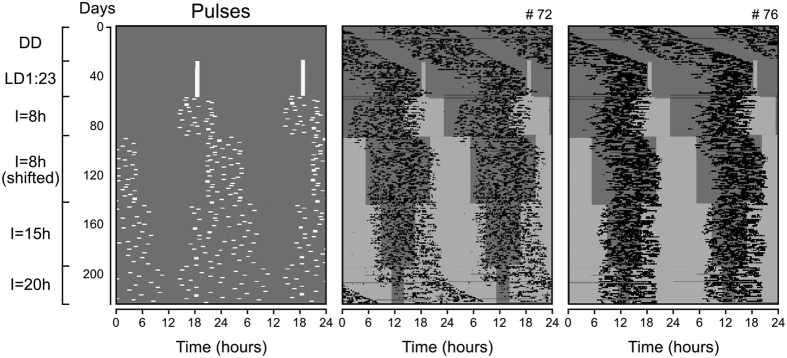
Synchronization of tuco-tucos’ circadian activity rhythms to regimens of 1 light pulse per day distributed at random times. Left graph: protocol indicating timing of the daily single 1-hour light pulses of intensity **L** = 1,000 lux (white marks), against the darkness (gray) background. The dispersion of pulses was progressively increased from the first to the last light regimen, as described on the left of the actogram. Middle and right graphs: actograms of the rhythms from two representative animals. Light-gray areas on the background represent the time interval “I” to which the single daily pulses were restricted. Activity times are marked in black. Actograms from all individuals are shown in Supplementary Fig. S1.

In the first experiment, we verified the limits to which the interval “**I”** could be widened and still promote 24-hour synchronization (Fig. 3). Tuco-tucos (N = 9, 6 males, 3 females) were initially left in constant darkness and their running-wheel activity rhythms free-ran with periods different from 24 hours, as illustrated by two representative animals (Fig. 3, DD). We then applied the sequence of single daily light pulses represented on the left panel of Fig. 3 (Pulses); each light pulse treatment lasted from 29 to 54 days.

In response to daily pulses at the same time every day, the rhythms assumed periods closer to 24 hours, after some transient cycles (Fig. 3, LD1:23). Next, the timing of the single daily light-pulses was randomized within an 8-hour time-window (Fig. 3, **I** = 8 h). Some transients were observed, but the rhythms attained synchronization to a 24-hour period. To confirm that the observed synchronization was due to photic entrainment and not to some uncontrolled environmental cycle, we delayed the pulse interval by 6 hours. The activity band was delayed accordingly, indicating that the pulses actively entrained the underlying circadian oscillator (Fig. 3, **I** = 8 h (shifted)). Period quantification of the rhythms from all animals confirms the maintenance of periods close to 24 hours upon exposure to the randomly timed light pulses within an 8-hour interval (Supplementary Fig. 2).

After an increase in the time window of pulses to 15 hours, the rhythms still sustained a 24-hour periodicity (Fig. 3, **I** = 15 h, Supplementary Fig. S2). When the pulse interval **I** was finally widened to 20 hours, the two representative animals presented different responses (Fig. 3, **I** = 20 h). Animal #72 expressed a rhythm with period greater than 24 hours, while animal #76 sustained the 24-hour rhythmicity. Numerical data from all individuals show a great increase in the variability of periods and an overall deviation from 24 hours (Supplementary Fig. S2). Of the 9 animals, 3 presented rhythms with periods far from 24 hours, 2 became arrhythmic and 4 remained with a 24-hour rhythmicity in this last regimen.

Our previous computer simulations predicted that, even though the circadian oscillator should remain entrained to 24 hours in regimens with large **I**^18^, the day-to-day phase variability should be increased as the light pulse times became more disperse. In our current experiment with tuco-tucos, however, we found no obvious tendency for higher phase variability in regimens of greater “**I**”, regardless whether using activity onsets, activity offsets or center of gravity as phase markers (Supplementary Fig. S3). Finally, periodogram analysis from rhythms associated with increased **I** did not show side-band periods^22^ that indicate gradual loss of entrainment (Supplementary Fig. S4).

### Entrainment under pulse regimens of decreasing dispersion

**Figure 4:**
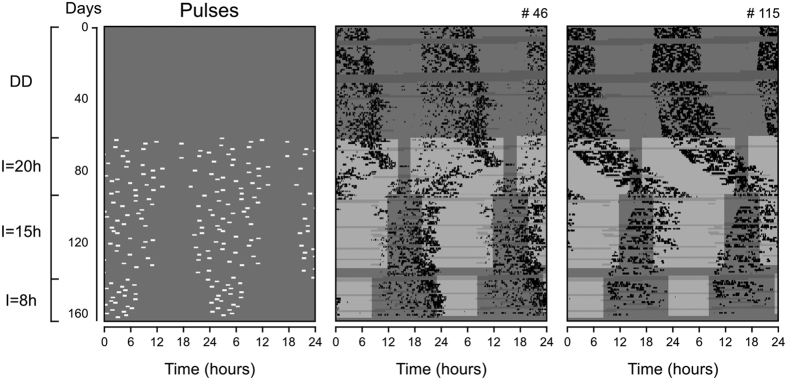
Effects of light-pulse regimens applied on tuco-tucos in a converse order relative to the first experiment. Light pulses were applied at an intensity **L** = 1,000 lux. For other specifications, see Fig. 3. Actograms from all individuals are shown in Supplementary Fig. S5.

We next tested whether the synchronization by randomly distributed light stimuli was dependent on the order of the light pulse regimens. In this sense, we repeated the last three light pulse regimens shown in Fig. 3, however, in the converse order, with 6 animals (3 males and 3 females). Three of the animals (#07, 60, 80) were reused from the first experiment, which finished 8 months earlier. Data from two representative animals are shown in Fig. 4. Both presented circadian rhythms in constant darkness (Fig. 4, DD). In the **I** = 20h regimen, the rhythms of most individuals attained periods different from 24 hours (Fig. 4, **I** = 20h) and only 1 presented stable 24-hour rhythmicity (Supplementary Fig. S2).

Upon transfer to the **I** = 15h regimen, the rhythm from animal #46 was apparently adjusted to 24 hours, with signs of relative coordination (Fig. 4, **I** = 15h). Animal #115 was either not prone to synchronization or was still in transients and would require more days to synchronize to the **I** = 15h regimen. Quantification (n = 3) returned periods closer to 24 hours upon transfer from **I** = 20h to **I** = 15h, confirming a tendency for synchronization (Supplementary Fig. S2). Three animals could not be followed in the transition to **I** = 15h due to data loss (#07, 60) or arrhythmicity (#80) (Supplementary Fig. S5), therefore, conclusions should be taken with caution.

In the **I** = 8h regimen, both representative animals had their rhythms adjusted to 24 hours with activity concentrated during the hours of “darkness”, when no light-pulse was presented (Fig. 4, **I** = 8h). Quantification of the rhythms from the whole group confirmed periods close to 24 hours in all the measurable individuals (Supplementary Fig. S2); two animals could not be analyzed at this regimen due to data loss.

### Entrainment under pulse regimens of different light intensity

Finally we asked how the strength of the light pulses (light intensity **L**) would influence the synchronization to our simplified light exposure model. Following the previous experiment, animals were immediately transferred to DD to start the new experiment; female #115 was not used in the analyses because of great data loss. They were thereafter exposed to pulses distributed at **I** = 15h with **L** = 100 lux and then to pulses at **I** = 15h with **L** = 1,000 lux.

**Figure 5:**
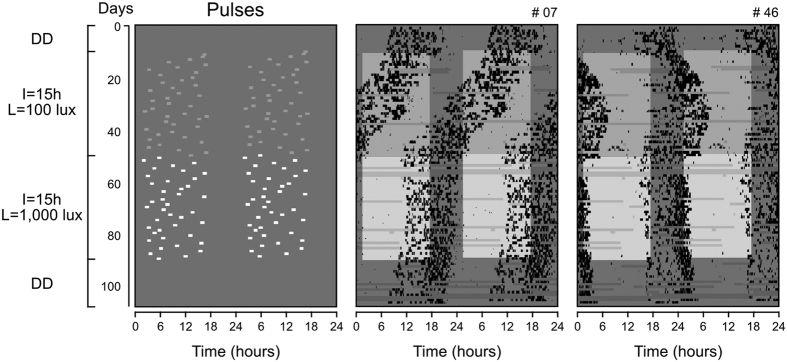
Effect of light intensity in synchronization to the random daily single light-pulse regimen. Two gray tonalities are used to represent the weak- (darker) and strong- (brighter) pulse regimens. For other specifications, see Fig. 3. Actograms from all individuals are shown in Supplementary Fig. S6.

Figure 5 shows the light pulses and the activity records from two representative individuals. In the **I** = 15h (**L** = 100 lux) regimen, neither of the representative animals was stably synchronized by this pulse regimen; animal #07 presented period shorter than 24 hour, while animal #46 was in relative coordination (Fig. 5, **I** = 15h **L** = 100 lux). Quantifications confirm that only 1 animal presented a 24-hour rhythm in this regimen (Supplementary Fig. S2).

Upon increasing light intensity to **L** = 1,000 lux (same intensity used in the previous experiments), the rhythms of both animals displayed periods very close to 24 hours (Fig. 5, **I** = 15h **L** = 1,000 lux). The quantified periods of all individuals confirm the tendency: 4 out of 5 lied within the 24-hour range in **L** = 1,000 lux (Supplementary Fig. S2). Upon release into DD, periods remained in the range of 24-hours, indicating period aftereffects of entrainment from the last pulse regimen (Fig. 5, DD; Supplementary Fig. S2). The distinct transient patterns shown in **I** = 15h (**L** = 1,000 lux) were replicated in computer simulations, using different light intensities for the simulated pulses (Supplementary Fig. S7, Supplementary simulations).

## Discussion

Our field and laboratory data reveal a simple and non-intuitive way of photic entrainment that, to our knowledge, has not been explored before experimentally. The results confirm and extend the predictions from computer simulations^18^ and strongly suggest that the process of entrainment is even more robust than previously known.

Synchronization is achieved when the light/dark cycle of the Earth forces a circadian oscillator to a 24-hour period. Two parallel conceptual models of photic entrainment have long coordinated our understanding of the mechanism behind this period-forcing process (Supplementary Fig. S8). The “parametric” (continuous) model proposes that the circadian oscillator is continuously driven by the light/dark cycle to achieve a 24-hour period. The “non-parametric” (discrete) model, on the other hand, proposes that the timing of the oscillator is reset by abrupt phase shifts, just like we kick a swing in discrete, periodic drives. In this model, light would not affect the oscillator throughout the entire duration of the light phase but especially during the transitions that occur at twilight times^23^.

The physical basis of these two modes has been simulated by limit-cycle oscillators driven either by continuous, harmonic forces^24^ or by impulsive, kicking forces^25^. The latter model relies on the time-dependent phase responses of circadian oscillators to light pulses, which are provided by species-specific Phase Response Curves (PRCs)^12^,^17^,^26^,^27^. It was not evident, however, how these theoretical constructions could account for entrainment in the “real world”, where each species in its habitat displays specific light exposure patterns.

It has been proposed that non-parametric mechanisms could reliably model the natural photic entrainment of nocturnal animals^7^,^28^,^29^, that presumably expose themselves to light briefly during dawn and/or dusk hours^30^. This proposition was supported by DeCoursey^31^ in her pioneering approaches to natural entrainment with flying squirrels in simulated laboratory burrows. The flying squirrels were allowed the opportunity to retreat to their burrows and expose themselves voluntarily to light and, indeed, they achieved entrainment by sporadic phase resetting of the circadian oscillator, at times of self light exposure.

Could a similar mechanism explain entrainment in diurnal organisms? Daytime activity presumably implies exposure to light during most of the activity phase; thus, an initial, non-parametric approach conceived symmetric phase resetting occurring at dawn and dusk^32^. However, the first field study on natural entrainment, using diurnal ground squirrels carrying light sensors, showed that these animals are not exposed at all to light during twilights^6^, favoring a more parametric-like approach for diurnal entrainment^33^.

One non-intuitive prediction of the non-parametric model is that few minutes or even a single second of light per day is sufficient for photic entrainment^34^,^35^. This has been successfully tested experimentally with single pulse T-cycle experiments, in which single light pulses are administered at a fixed phase within a cycle (in the same time of day, for 24-hour cycles)^35^,^36^ The phenomenon can also be simulated by physical oscillators of the limit-cycle type, which are synchronizable by periodically-kicking forces^25^.

Our computer model of photic entrainment^18^ provided an unexpected and even less intuitive prediction than the T-cycle regimens: entrainment could be sustained by a single daily light pulse administered at randomly changing times within a daytime interval. Computer simulations also predicted that, for our light pulse duration and intensity, synchronization would prevail even if this daytime interval achieved surprisingly wide lengths such as 14 hours.

The present experimental tests inspired by our in silico mathematical model confirmed that the simplified light regimen is sufficient to sustain entrainment also *in vivo*. In nature the studied *Ctenomys* species in Anillaco, Argentina, never experiences photophases greater than 14 h 53 min (civil twilight times available at http://www.arachnoid.com/lutusp/sunrise, accessed on March 14^th^, 2016), which is less than the 15 hours that successfully sustained entrainment in the lab. This situation is a simplification of light exposure patterns in the field for a subterranean animal and in particular for tuco-tucos, which are definitely exposed to light at several random times per day, according to our current light-logger data. Therefore, simulations, laboratory and field data indicate that photic entrainment is a strong component of field entrainment in subterranean tuco-tucos.

It has long been suggested that parametric and non-parametric models should be integrated for a complete understanding of photic entrainment^37^. Peterson^38^ posed this question while observing the “sluggish” behavior of the *Drosophila* circadian oscillator when released from constant light to darkness or the reverse way. This concept of sluggish dynamics was better formalized in Abraham *et al*.^39^, where the “relaxation rate” was brought to light as a parameter that determines how fast a limit-cycle’s amplitude is recovered after exposure to light stimuli^40^. This concept challenges and complements the instantly recovering oscillations presumed by the non-parametric model.

We propose that the slow relaxation rate of circadian oscillators underlies their ability to recognize a 24-hour period out of randomly distributed light pulse times. Each day the light stimulus impinges a phase-shift on the oscillator, but the shift occurs only after transient slow changes in amplitude^22^,^38^,^39^,^41^. If the amplitude were instantly recovered our random pulse regimens would presumably cause equally random back and forth changes in the phase of the circadian oscillator, possibly failing to keep the phase stable within a 24-hour day. This effect was not displayed in our entrainment patterns – as shown by the equal phase dispersion of activity under different regimens (Supplementary Fig. S3).

Instead, the transient phase shifts that occur at each new light pulse result in the overall sluggish process that resembles a parametric, continuous adjustment, even though there is no continuous light. Thus, although our studies of synchronization under this peculiar, random light regimen were not meant to discern parametric from non-parametric models, they reveal that photic entrainment is achieved by the contribution of both components. The key to this integration lies in the fact that there is no “pure” non-parametric synchronization, because circadian limit-cycle oscillators^40^ seem to display non-instant recovery of their amplitude.

It has been shown that entrainment of a free-running circadian oscillator occurs only for a specific range of period and light intensity combinations of the light/dark cycle which set the “range of entrainment”^22^,^39^,^41^. In our “random pulse” paradigm, we have fixed the period of the desired entrainment to 24 hours and have instead manipulated the dispersion length **I** (Figs 3 and 4) or the intensity **L** of light pulses (Fig. 5). We then proceeded to see if the concept of range of entrainment could also be established for these particular parameters, **I** and **L**.

For a fixed light intensity **L** of 1,000 lux, entrainment was lost after **I** was gradually increased to 20 hours, as predicted by our computer simulations^18^. We then converted the direction of **I** change, from a gradual increase to a decrease, to assure that the 24-hour period synchronization at each new **I** step of the protocol was not dependent on the period aftereffects of previous entrainment^35^,^42^. Indeed, entrainment was similar (Figs 3 and 4, S2), regardless of the direction of the change in **I**, with most periods farther from 24 hours in **I** = 20 h and closer to 24 hours in **I** = 15h. In **I** = 8h, all animals remained within the 24-hour range.

On the other hand, for a fixed **I**, random pulse regimens that sustained entrainment for the first light intensity lost this ability when this parameter **L** was weakened, both in our experiments (Fig. 5) and in computer simulations (Supplementary Fig. S7, Supplementary simulations). As it occurs with the period in the range of entrainment, our data suggests that the “threshold **I** for entrainment” was modified with **L**. The regimen **I** = 15h was within the range of entrainment when **L** was higher, however, outside this limit for weaker intensities.

Granada *et al*.^22^ have shown that close to the border of the range of entrainment, mathematical oscillators tend to assume a zig-zag-like pattern in the actograms, which are quantified by side-band periods in the periodograms. Similarly, in our computer studies with randomly distributed light pulse regimens^18^, mathematical oscillators close to the “threshold **I** for entrainment” also acquired a zig-zag pattern, with great phase-variability along consecutive days and side-band periods in the periodograms. In the experimental work with tuco-tucos, we did not detect side-band periods, most probably because unlike in those simulations, what we observe here is the overt activity rhythm, not the state variable of the oscillator^43^ and this may make detection of these side bands more difficult.

Finally, it would be interesting to see how a system of weakly or strongly coupled oscillators^44^ would differ in their responses to a random pulse regimen. This is a complex and intriguing question, since coupling force changes both the range of entrainment and the relaxation rate of the emergent oscillatory system^39^.

Photic entrainment studies are usually based on model species and laboratory experiments. Field information from unconventional, wild species can add to these studies, because each species presents a particular temporal pattern of light exposure, revealing extreme photic conditions that may inspire refinements or reelaborations of theoretical entrainment models^31^,^45^,^46^. In this context, here we present a new, random pulse paradigm that provides less periodic timing information, than the single pulse T-cycle, and which was inspired by our natural measurements of daily light exposure patterns in a subterranean rodent. Previous computer simulations of this paradigm had predicted an unexpected robustness of entrainment to daily light stimuli at irregular phases and we successfully tested these predictions experimentally. Furthermore, we suggest that this paradigm presents a route for integrating “parametric” and “non-parametric” components of photic entrainment. Our field, laboratory and computer simulation approaches come together to uncover new properties of circadian oscillators.

## Methods

### Ethics statement

Trapping and laboratory protocols were approved and authorized by the Legal and Technical board (*Oficina de Técnica Legal*) of the Environmental Department of La Rioja (*Secretaria de Ambiente, Ministerio de Producción y Desarrollo Local*), permit number 062–08. They were also approved by the CEUA from the Institute of Biosciences, University of São Paulo (Protocol 152/2012) and by the CICUAL (Comité Institucional para el Cuidado y Uso de Animales de Laboratorio) from the *Facultad de Ciencias Veterinarias*, *Universidad de La Plata* (Protocol 29-01-12). Every procedure in this study followed the guidelines of the American Society of Mammalogists for animal care and handling^47^.

### Animal trapping

Tuco-tucos were obtained from a natural population, in the Monte Desert^48^, east of the town of Anillaco, La Rioja, Argentina (28°48′S; 66°56′W; 1,350 m). Animals were captured with PVC live-traps similar to the ones described in^49^, placed at freshly tapped burrow entrances and checked every 2 hours.

### Semi-Natural Enclosures and Light Exposure Recordings

Three outdoor enclosures were built in an area that is naturally occupied by wild tuco-tucos. Two enclosures measuring 12m × 6m were protected by wire mesh 1.5m aboveground and 1m underground, and a horizontal 20cm barrier in the inner perimeter at the bottom. The third enclosure measured 10m × 5m and was protected with wire mesh on top and sides (1.2m above-ground and 1m underground). The size of the enclosures was based both on the home-range determined for *C. talarum*^50^ and on our telemetry-based estimation of the summer home range of the *Ctenomys* species found in Anillaco.

A meteorological station (Onset Computer Corporation, Bourne, MA) located at the site of the enclosures allowed constant recording of ambient temperature. Underground temperature was also continuously measured at a fixed 60-cm underground location inside the burrow using HOBO data loggers U10/003 (Onset Computer Corporation, Bourne, MA). Average temperatures were similar in the two years of recording – July 2014 max/min ambient temperatures: 18.9 ± 5.6 °C/4.7 ± 3.8 °C; max/min underground temperatures: 13.8 ± 1.2 °C/ 13.4 ± 1.1 °C; July 2015: max/min ambient temperatures: 19.4 ± 5.4 °C/ 5.2 ± 5.1 °C max/min underground temperatures: 14.6 ± 1.1 °C/ 13.6 ± 3.6 °C.

A total of 11 tuco-tucos were released in the field enclosures, one at a time, in the two consecutive years. Of those, we were able to recover the records of light exposure from 8 individuals (4 males and 4 females, average weight 175 ± 44g). Before field recordings, animals were maintained in the laboratory, next to an open window, exposed to the natural light/dark cycle. Due to other experiments, they had implanted i-button temperature sensors and had remained in the lab for 1 to 5 months, before release into the enclosures. They were fed daily, at random times, with various items such as carrots, sweet potatoes, rabbit chow, sunflower seeds, oatmeal and lettuce.

On the day of release, individuals were anesthetized with isoflurane (1–1.5min, 3.5–4%, 4 L.min-1 O_2_) and received light loggers affixed to neck collars (package weight <5g; Intigeo loggers C65, W50, W65; Migrate Technology, Cambridge, UK). The collars were made of zip ties covered by silicone tubing. Light-loggers were set to detect illuminance every minute in the range of 0.3 to 19,000 lux (animals #153, 154, 155, 180, 184, 188) or 1 to 74,000 lux (animals #183, 185). Recorded data consist of a sequence of the highest values within each 5-minute interval.

Three animals were released at a time, one in each enclosure, in the winters of 2014 (July) and 2015 (July and August). Upon release, each animal readily excavated its own burrow system. No extra food was provided besides the natural vegetation. After 5 to 12 days in the enclosures, recapture was achieved using the same PVC tube traps described above and collars were removed immediately in the laboratory.

### Laboratory Housing and Experiments

For the laboratory experiments, 12 tuco-tucos in total (7 males and 5 females, average weight of 178 ± 32g) were captured, as described above, and transferred to individual cages (length × width × height = 53 × 29 × 27cm) with running wheels (diameter 23 cm, width 10 cm, distance between bars 10 cm). They were kept in an isolated room with controlled light and temperature (T = 24 ± 1 °C) and were fed daily, as explained above. Cages were cleaned weekly. Running wheel turns for each animal were counted at 5-minute intervals by the Simonetta System (Universidad Nacional de Quilmes, Buenos Aires), to continuously record the animal’s activity/rest rhythm.

Red incandescent light bulbs remained turned on continuously, at low illuminance (<5 lux) to allow proper cleaning and feeding during darkness conditions. White fluorescent bulbs were used to define the conditions of “light” and “dark”, superimposed to the background red light. The fluorescent light was regulated with a field light meter (TMI-201, Tenmars Electronics CO., Taiwan) to provide the two different illuminance levels, at the height of cage lids (**L** = 100 or 1,000 lux). Light schedules in each experiment were programmed weekly into digital timers DNI-6610 (Dani Condutores Elétricos Ltda., São Paulo), which automatically promoted the light/dark changes.

### Experimental light/dark schedules

Light pulses were applied once a day at random times, restricted to a distribution interval **I** of 8, 15 or 20 hours, as indicated in each experiment. The random pulse times were generated in Microsoft Excel (2007) with the function RANDBETWEEN. Each light pulse consisted of turning the fluorescent lights on for 1 hour at the specified light intensity. In the LD1:23 regimen, the 1-hour light pulses were delivered at the same time every day. During constant darkness (DD) only the dim background red light was left on.

### Entrainment under random pulses with increasing dispersion

9 tuco-tucos (6 males, 3 females) were exposed to daily light pulses in the following sequence of treatments: constant darkness (DD); light/dark cycle with 1 hour of light and 23 hours of darkness per day (LD1:23); daily light pulses within an 8-hour interval (**I** = 8h); daily light pulses within 8 hours, but delayed in 6 hours relative to the previous regimen (**I** = 8h (shifted)); daily light pulses within a 15-hour interval (**I** = 15h); daily light pulses within a 20-hour interval (**I** = 20 h). Each light pulse consisted of turning the lights on for 1 hour at **L** = 1,000 lux.

### Entrainment under random pulses with decreasing dispersion

6 animals (3 males, 3 females) previously free-running in DD were exposed to the sequence **I** = 20h, **I** = 15 h and **I** = 8h. Light pulses consisted of 1 hour of lights on at **L** = 1,000 lux.

### Entrainment under random pulses with different light intensities

5 animals (3 males, 2 females) were exposed to two pulse regimens with the same distribution window “**I**”, but with different light intensities, in the following sequence of treatments: DD, **I** = 15h (1-hour pulses of **L** = 100 lux) and **I** = 15h (1-hour pulses of **L** = 1,000 lux).

### Data analysis

Actograms and histograms for field light exposure data were built in Microsoft Excel. To build each actograms, we considered that a light exposure episode happened whenever the light level exceeded the basal (lowest) value of each individual record. For histograms, the light exposure episodes were summed in 15-minute intervals, to reveal the overall distribution of light exposure throughout the day, excluding the days of release and recapture.

Laboratory data was visually analyzed in actograms made with the software El Temps (A. Díez-Noguera, Universitat de Barcelona, 1999). Statistical quantification of the rhythms’ periods was done using the chi-square periodogram with 0.05 significance level and 5-minute period precision^51^, in the software Clocklab (Actimetrics, Wilmette, USA). For each experimental regimen, data from the last 15 days were used for period determination. The rhythm was considered synchronized when its period lied within the 24 h ± 10 min interval. Phase was determined through activity onsets, offsets and “center of gravity” (CoG)^52^ from the last 15 days of each experiment. Phase dispersion was calculated from the standard deviation of these reference phases, only when synchronization to 24 hours was achieved.

## Additional Information

**How to cite this article**: Flôres, D. E. F. L. *et al*. Entrainment of circadian rhythms to irregular light/dark cycles: a subterranean perspective. *Sci. Rep*. **6**, 34264; doi: 10.1038/srep34264 (2016).

## Supplementary Material

Supplementary Information
