# Supplementary material for: Entrainment of circadian rhythms to irregular light/dark cycles: a subterranean perspective
Source: Sci Rep. 2016 Oct 4;6:34264. doi: 10.1038/srep34264 (PMC5048425; doi:10.1038/srep34264)
Supplement: Supplementary Information [file srep34264-s1.pdf]

[Supplementary Information]

Entrainment of circadian rhythms to irregular light/dark cycles: a subterranean perspective

Danilo E. F. L. Flôres, Milene G. Jannetti, Veronica S. Valentinuzzi, Gisele A. Oda

Supplementary simulations

To help the interpretation of the distinct patterns observed in Figure 5, computer simulations were performed, using a model limit-cycle oscillator developed by Pavlidis and Pittendrigh (Pittendrigh et al., 1991), which is defined by the following equations:

$$\frac{dR}{dt} = R - cS - bS^2 + d - L + K$$

$$\frac{dS}{dt} = R - aS$$

In these equations, R and S are state variables and letters a, b, c and d are four parameters that determine the oscillator configurations. We used a standard configuration (a=0.85, b=0.3, c=0.8, d=0.5), already employed in Flôres et al.[18]. K is a small nonlinear term (K = 1/[1 + 100R2]) formulated by W.T. Kyner, to prevent the variable R from achieving negative values. Simulated locomotor activity occurred every time the S variable rose above a threshold value, which we set to two thirds of the maximum amplitude of this variable. L represents the light level: a 1,000 lux light pulse, for instance, is mimicked by square-wave changes of L from baseline 0 to amplitude 1.1 (arbitrary unit) for the duration of the pulse. We used the Euler method for numerical integration, with 1,000 integration steps per 24 hour day. Simulations were performed using the *NeuroDynamix* software (www.neurodynamix.net).

We verified synchronization patterns of the model oscillator when exposed to daily 1-hour light pulses, whose daily timing changed randomly within a fixed interval **I**=15h. The times of light pulses were the same used in the experiment reported in Figure 5.

To check the response to weak or strong pulses on the long term, we first exposed the model oscillator to pulses with fixed amplitude throughout the whole simulation (Supplementary Fig. S7A, B, C). When subjected to weaker pulses, the oscillator was either not synchronized to 24 hours during the 80 days of pulses (Supplementary Fig. S7A) or it took several cycles (>60) to finally reach synchronization (Supplementary Fig. S7B). On the other hand, exposure to strong pulses readily synchronized the oscillator to 24 hours, within 30 days (Supplementary Fig. S7C).

At last, we repeated the protocol from the experiment reported in Figure 5, with two different pulse intensities in sequence (Supplementary Fig. S7D). The light pulses of the first 40 days had a lower light intensity (arbitrary value 0.13) while the light pulses of the last 40 days had the highest light intensity (arbitrary value 1.1). As can be seen from the figure, stable entrainment was only achieved by the stronger light pulse regimen.

Supplementary Reference

Pittendrigh CS, Kyner WT, Takamura T (1991). The amplitude of circadian oscillations: temperature dependence, latitudinal clines, and the photoperiodic time measurement. J Biol Rhythms 6: 299–313

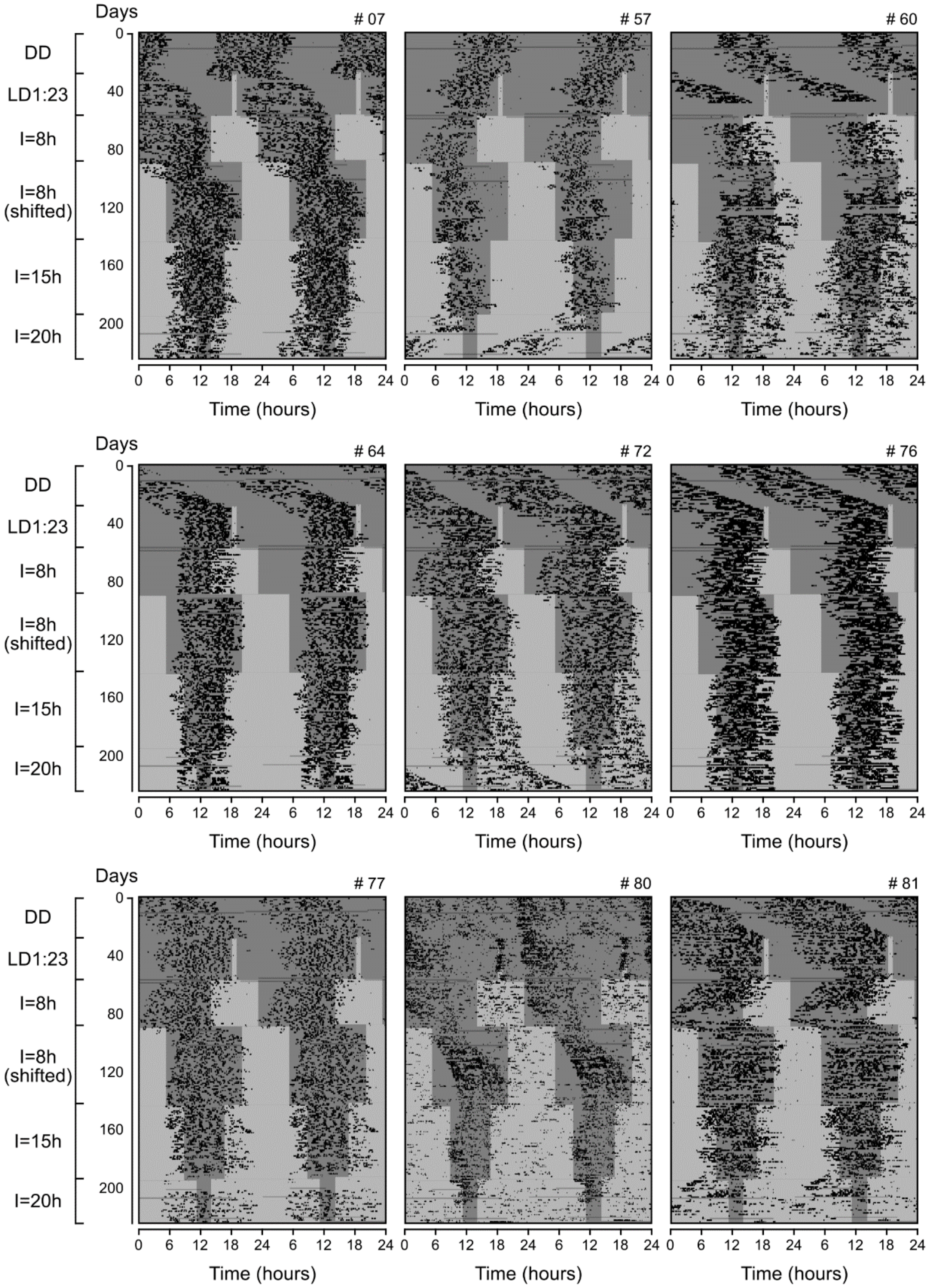

Figure S1. All actograms from the experiment with pulse regimens of increasing dispersion (Figure 3).

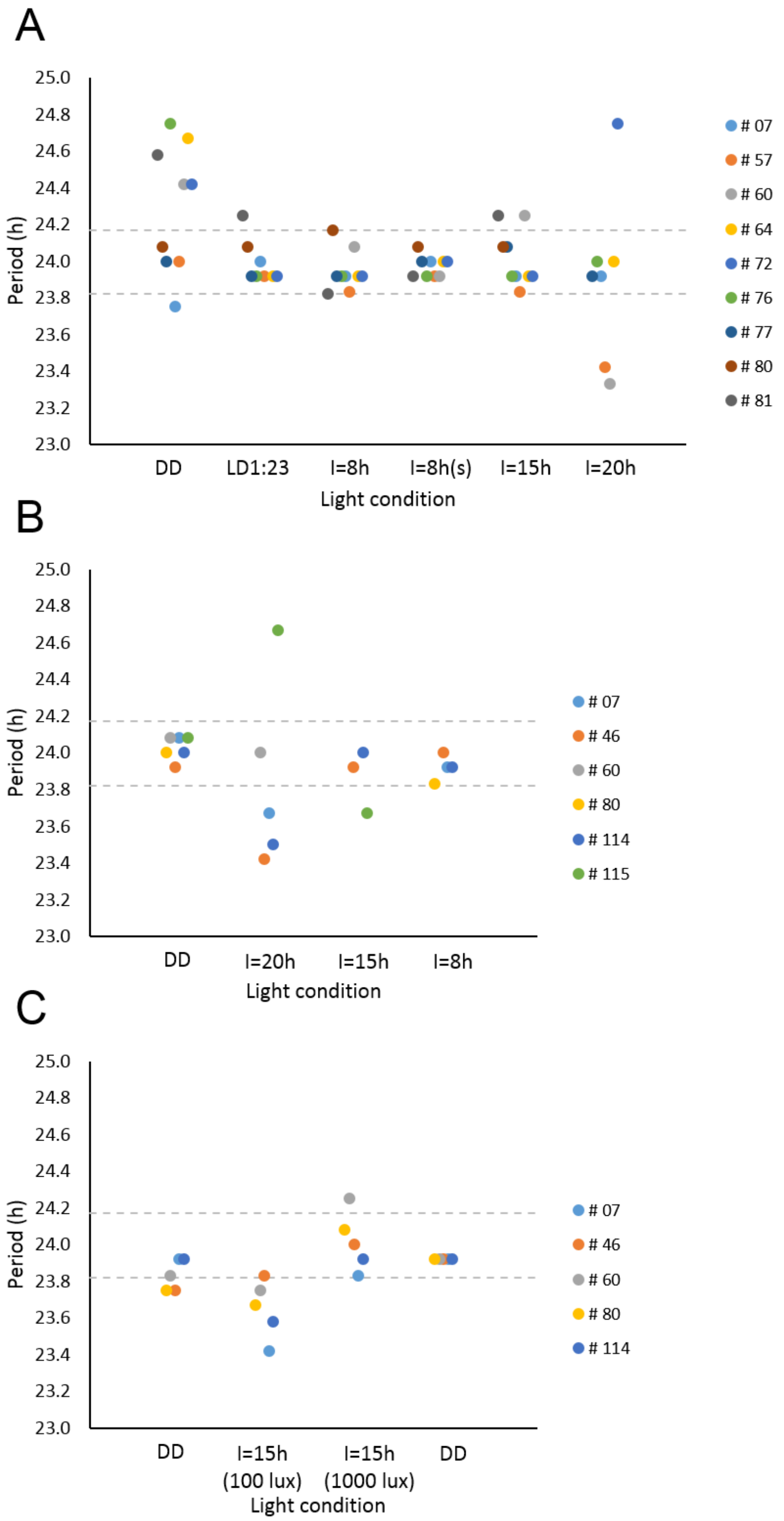

Figure S2. Period quantifications for each light regimen applied in the laboratory. A: Pulse regimens of increasing dispersion (Figure 3). B: Pulse regimens of decreasing dispersion (Figure 4). C: pulse regimens of different light intensity (Figure 5). Horizontal dashed lines indicate the 24 hour ± 10 minute interval.

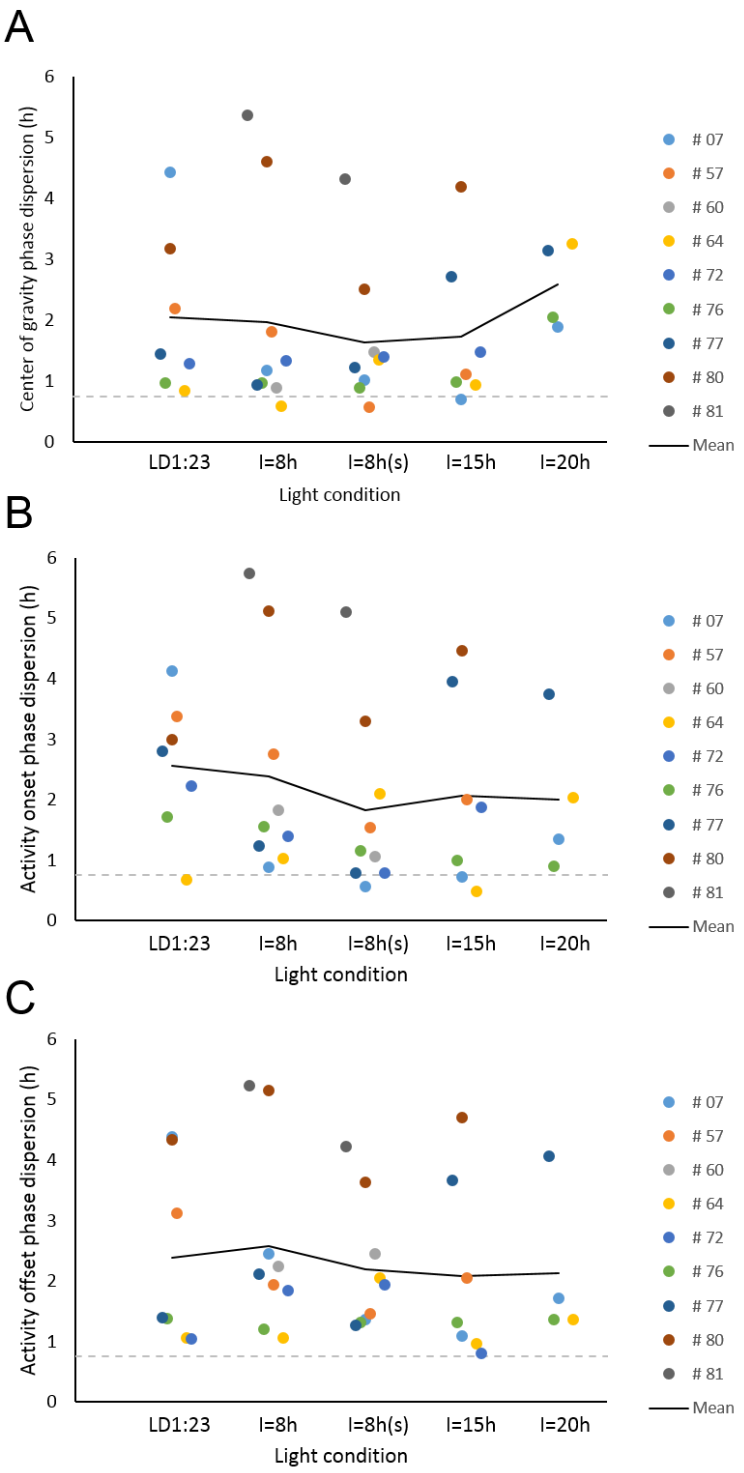

Figure S3. Phase variability of activity rhythm in the experiment with pulse regimens of increasing dispersion (Figure 3). The phase variability was based on different reference phase markers: (A) center of gravity (CoG), (B) activity onsets, (C) activity offsets. A dashed horizontal line indicates the maximal inherent phase dispersion that is unavoidable for rhythms with periods that deviate (± 10 minute) from 24 hours. In each graph, a solid line connects the average phase dispersion at the different light conditions.

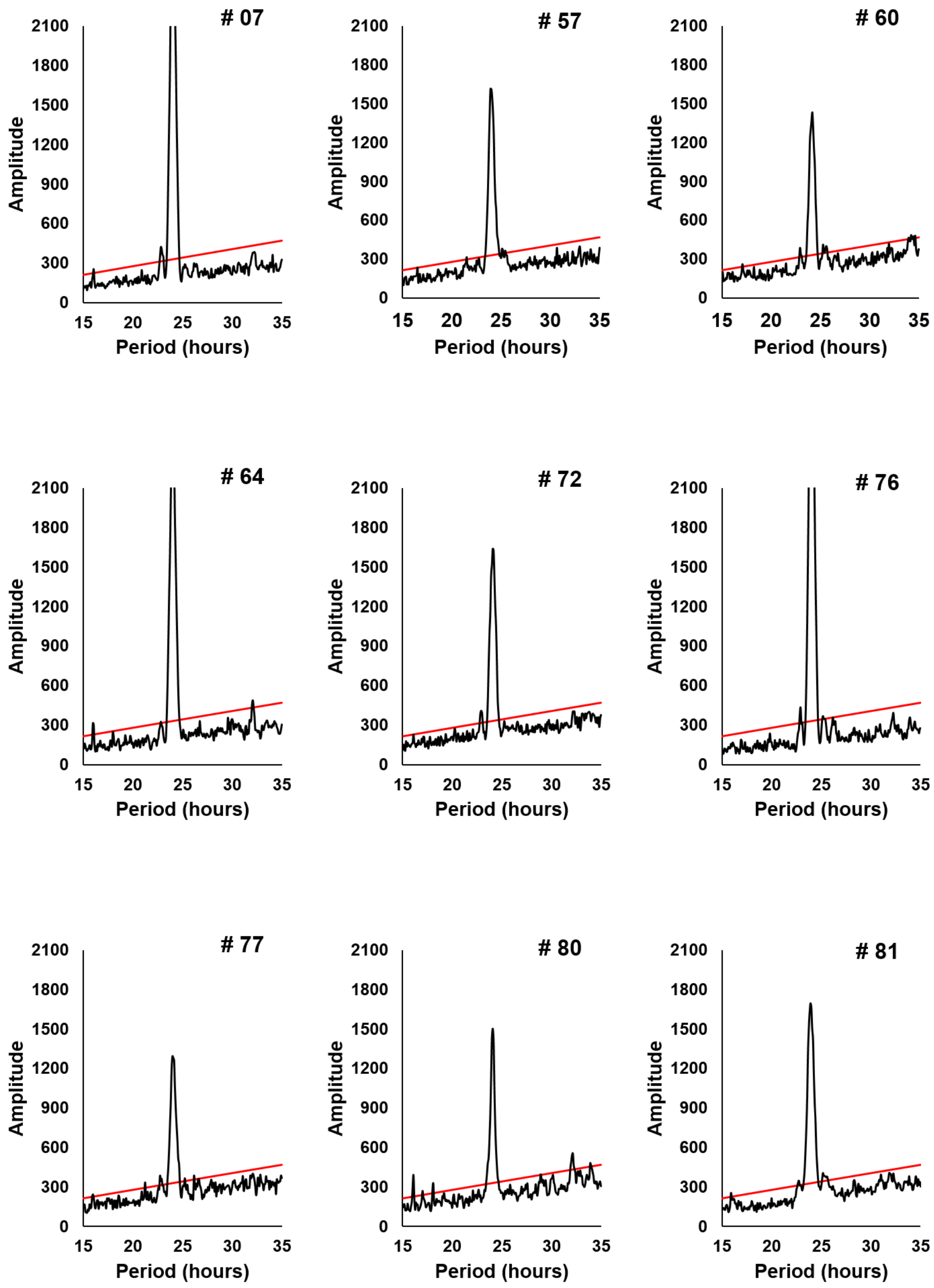

Figure S4. Period analysis of the rhythms from Figures 3 and S1, in the regimen I=15h. Each periodogram graph depicts the amplitude (significance) of the tested periods within the chosen range (15-35 hours), revealing the most probable (higher amplitude) period in the data, as well as potential side-band periods.

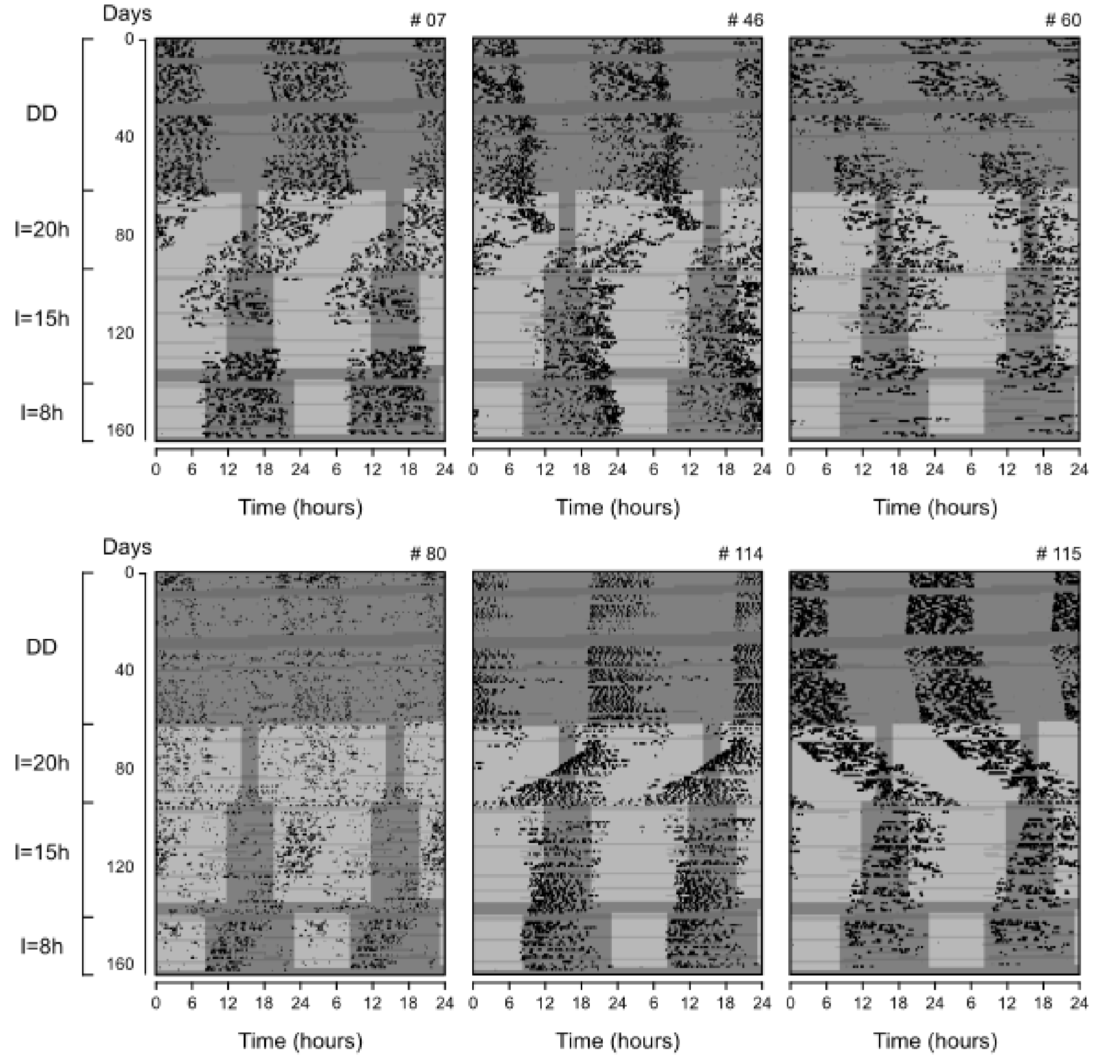

Figure S5. All actograms from the experiment with pulse regimens of decreasing dispersion (Figure 4).

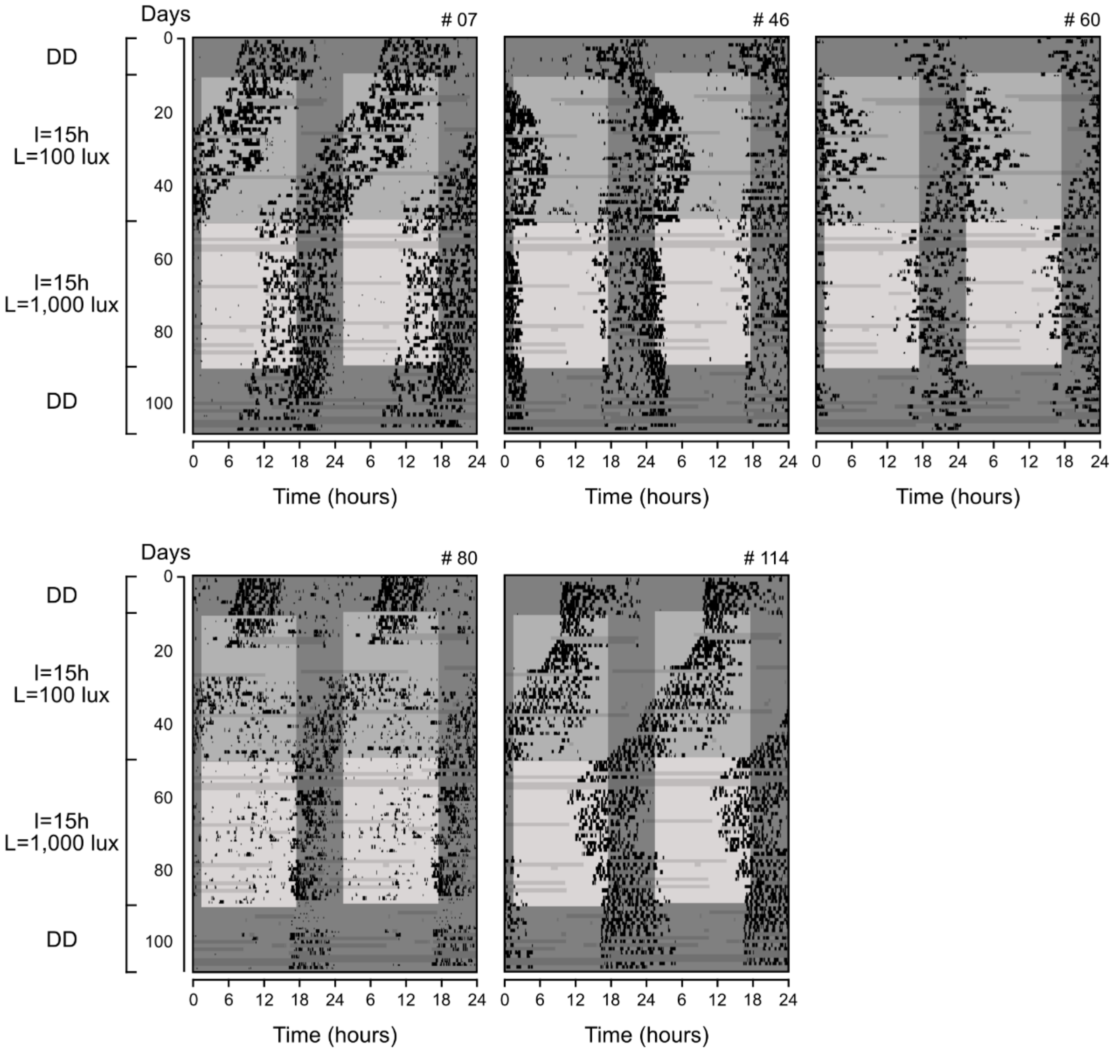

Figure S6. All actograms from the experiment with pulse regimens of different light intensities (Figure 5).

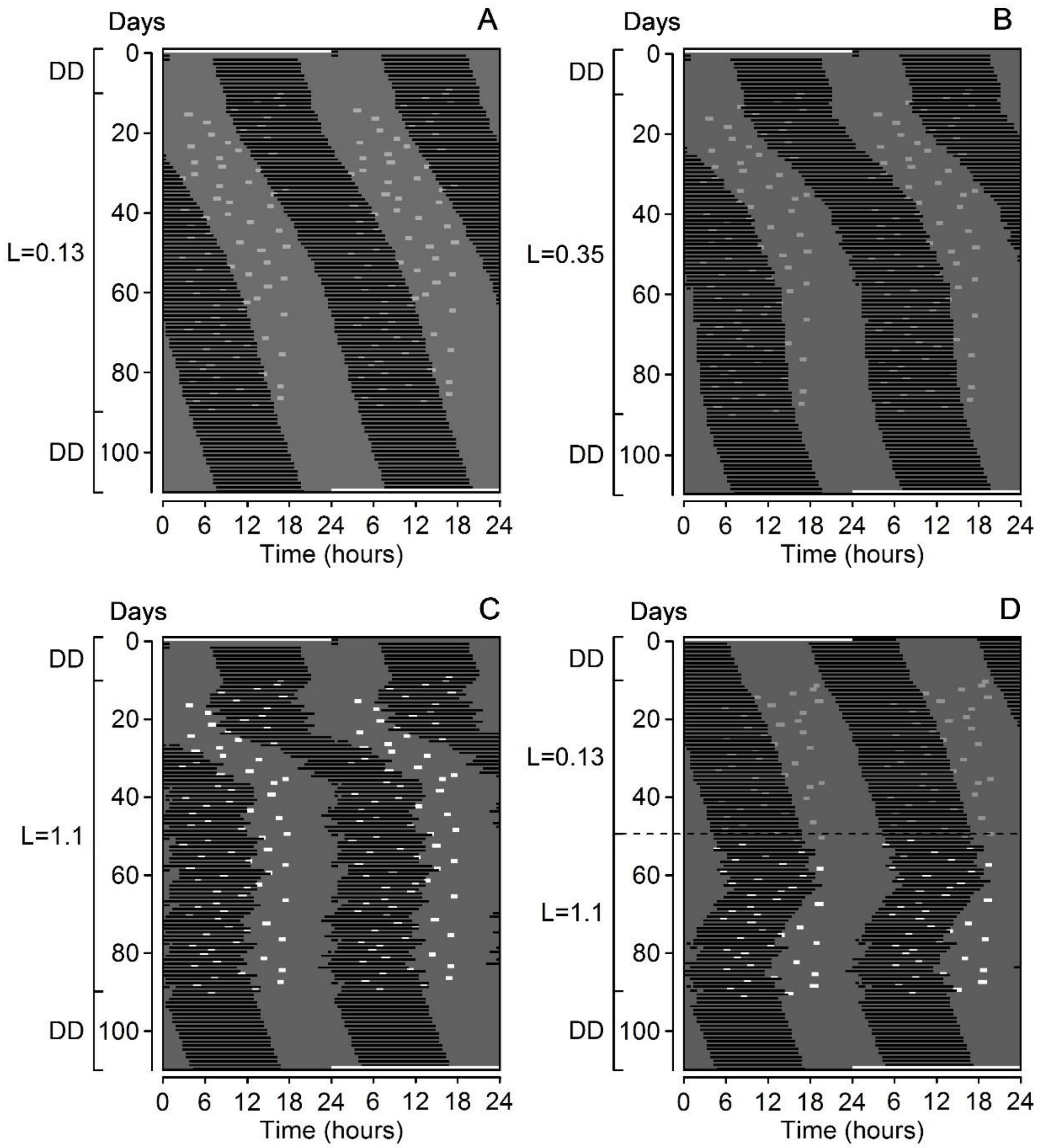

Figure S7. Computer simulations of a limit-cycle oscillator under the random pulse regimens of 1 light pulse per day and fixed interval I=15h. A) Weak light intensity (L=0.13); B) Intermediate light intensity (L=0.35); C) Strong light intensity (L=1.1); D) Combination of 40 days under weak (L=0.13) followed by 40 days under strong (L=1.1) light intensities, as in Figure 5. Light intensities are defined in arbitrary units. White and Black bars indicate light pulse and activity times, respectively. Oscillator parameters: a=0.85, b=0.3, c=0.8, d=0.5.

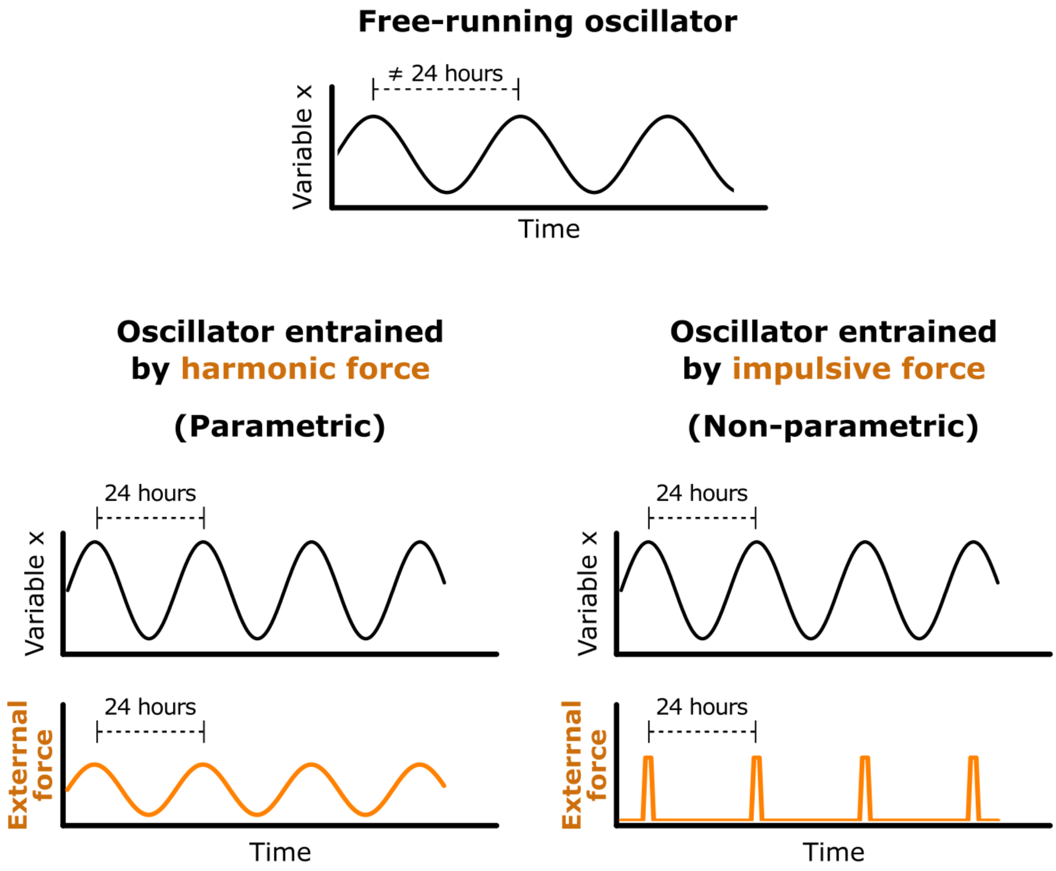

Figure S8. Two modes of entrainment. A free-running circadian oscillator (upper figure) has an inherent period that is different from 24 hours. Conceptually the oscillator period can be entrained to the 24 hours of the environment either by a harmonic, continuous force (lower left), or by an impulsive, discrete force (lower right). These two models are used to explain the parametric and non-parametric entrainment, respectively.
